# Rituximab Used for the Treatment of Nonuremic Calciphylaxis: A Complication of Prolonged Steroid Use in Lupus Nephritis

**DOI:** 10.7759/cureus.26516

**Published:** 2022-07-02

**Authors:** Cameron Kahn, Sukhraj Singh, Reshmi Mathew, Laurie A Ramrattan, Ibraheem J Mohammed, Reeba Omman

**Affiliations:** 1 Medicine, University of Florida College of Medicine – Jacksonville, Jacksonville, USA; 2 Rheumatology, University of Florida College of Medicine – Jacksonville, Jacksonville, USA; 3 Internal Medicine, University of Florida College of Medicine – Jacksonville, Jacksonville, USA; 4 Pathology and Laboratory Medicine, University of Florida College of Medicine – Jacksonville, Jacksonville, USA; 5 Pathology, University of Florida College of Medicine – Jacksonville, Jacksonville, USA

**Keywords:** chronic cutaneous lupus erythematosus, pyoderma gangenosum, skin necrosis, chronic corticosteroid use, nonuremic calciphylaxis, treatment of calciphylaxis, calciphylaxis, sle and rheumatoid arthritis, rituximab therapy, lupus panniculitis

## Abstract

Nonuremic calciphylaxis (NUC) is a rare and debilitating form of panniculitis. NUC is associated with a high mortality rate within the first year of diagnosis. Connective tissue diseases account for a small fraction of the reported cases. However, there have also been reported cases of patients developing NUC while on treatment with chronic corticosteroid immunosuppressive therapy. The pathophysiology of NUC is still not fully established. Several risk factors including underlying diseases, obesity, female gender, and medications have been associated with the development of NUC. The diagnosis remains challenging due to the condition's similarities with other forms of panniculitis. The gold standard for diagnosis is a tissue biopsy showing calcifications within the medial layer of arterioles and the presence of microthrombi with surrounding necrosis. The treatment for NUC has not advanced much in recent years and focuses on the management of the underlying condition, wound care, and treating any superimposed infection. Treating superimposed infections remains important as most of the associated mortality from NUC occurs due to sepsis. We describe a case of a young woman with lupus nephritis who developed NUC while on prolonged corticosteroid therapy. She did not respond to several immunosuppressive agents and was ultimately treated with rituximab, a monoclonal antibody against CD20 antigen, as salvage therapy.

## Introduction

Calciphylaxis is a rare condition characterized by the emergence of nonhealing skin ulcers secondary to arterial calcification and thrombosis; it is typically diagnosed in patients with end-stage renal disease (ESRD) on hemodialysis. When it develops in patients without ESRD, it is called nonuremic calciphylaxis (NUC). NUC is a rare and debilitating form of panniculitis. Connective tissue diseases account for 11.1% of reported cases [1], and even fewer cases are reported in patients already on chronic corticosteroid immunosuppressive therapy. The proposed pathophysiological mechanism points to an imbalance of calcium and phosphate that exceeds their solubility, causing them to deposit within the medial layer of small vessel walls [2]. This causes fibrosis and proliferation of endothelial cells, termed subintimal fibroplasia [2]. Regardless of the underlying cause, mortality due to NUC has been reported to be upward of 50% within the first year of diagnosis [1]. The treatment for NUC predominantly focuses on the management of the underlying condition, wound care, and treating superimposed infections. There is no case in the existing literature where B-cell depleting therapy, such as rituximab, is used to treat NUC. We describe an interesting case of NUC in a patient with systemic lupus erythematosus (SLE) on prolonged corticosteroids, who, after failing several immunosuppressive agents, was ultimately treated with rituximab salvage therapy with an excellent response.

The abstract of this study was previously presented at the American Federation for Medical Research (AFMR) 2022 Southern Regional Meeting on February 11, 2022.

## Case presentation

**Figure 1:**
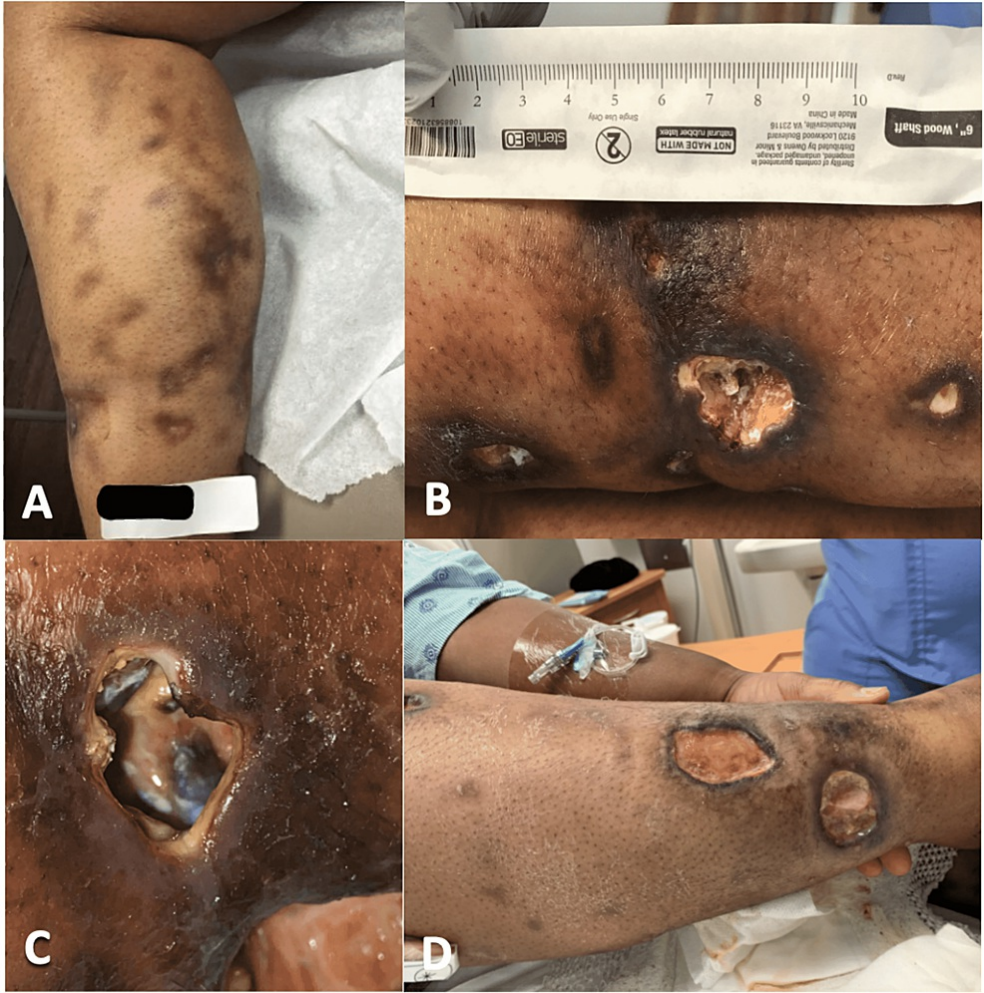
Progression of leg ulceration Pictures A-D show the progression from initial presentation (A) through the various failed treatments (B, C, D). Pictures C and D depict the lesions prior to initiating rituximab

A 32-year-old female with a past medical history of SLE, lupus nephritis Class II and V, interstitial lung disease (ILD), non-ischemic cardiomyopathy (NICM), and obesity presented with multiple painful leg ulcers. The patient had first noticed nodules on her right lower extremity a few months prior, which had progressively become more painful over several weeks. The ulcers exhibited indurated, hyperpigmented lesions of varying sizes that were extremely tender to touch (Figures 1A-1D).

**Figure 2:**
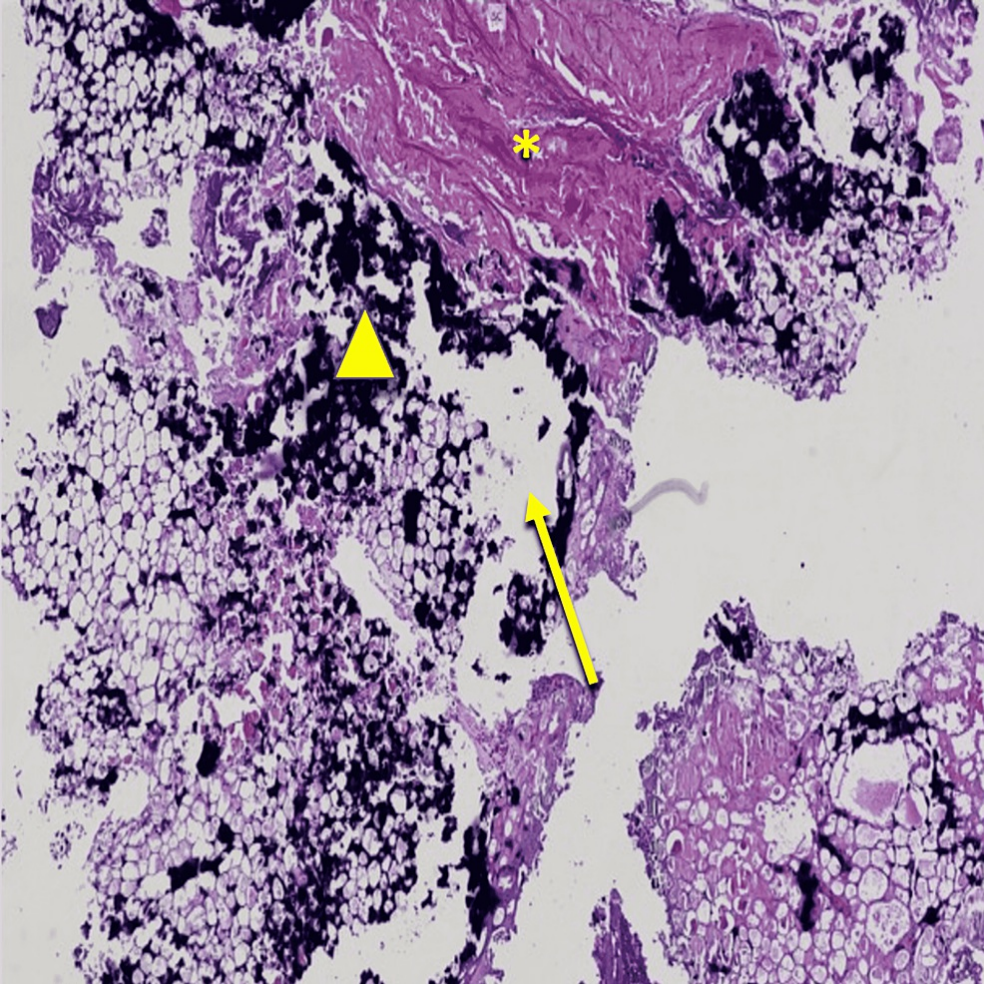
Histopathological examination showed cutaneous ulceration (arrow), fibrin thrombi (*) in dermal vessels, and calcium deposits in subcutaneous tissue (triangle)

The patient did not exhibit any other active manifestations of her connective tissue disease. Laboratory investigations showed normal renal function, calcium, phosphorus, vitamin D, parathyroid hormone (PTH), and complement levels. There were no elevations of anticardiolipin antibody, beta-2-glycoprotein antibody, or lupus anticoagulant. However, the patient had a chronically elevated anti-double-stranded DNA antibody despite immunosuppression with azathioprine 2 mg/kg daily and prednisone 10 mg daily. Of note, the patient had been intolerant to hydroxychloroquine (HCQ) due to central vision loss suspected to occur from retinal thinning. She also had previous intolerance to mycophenolate mofetil due to an allergic rash. The patient underwent a punch biopsy (4 mm) that revealed poor cell interface and vacuolar dermatitis with dermal mucin and sclerosis, suggestive of lupus profundus. The prednisone dose was increased to 1 mg/kg daily; however, the ulcers rapidly progressed despite additional immunosuppression and wound care. Dapsone was added to her regimen, but the ulcers continued to progress. The patient reported an increase in the size of her lower extremity ulcers and increasing pain with loss of skin and subcutaneous tissues. She was referred to the hospital as the ulcers continued to progress despite aggressive wound care, multiple courses of antibiotics, and an increased dosage of immunosuppressive medications. Ultimately, a second deep tissue open surgical biopsy was obtained to reassess the disease process. This biopsy revealed cutaneous ulceration, dermal vessels with fibrin thrombi, neutrophilic inflammation, and calcium deposits in the subcutaneous tissue. It also showed foci of fat necrosis with possible saponification (Figure 2).

**Figure 3:**
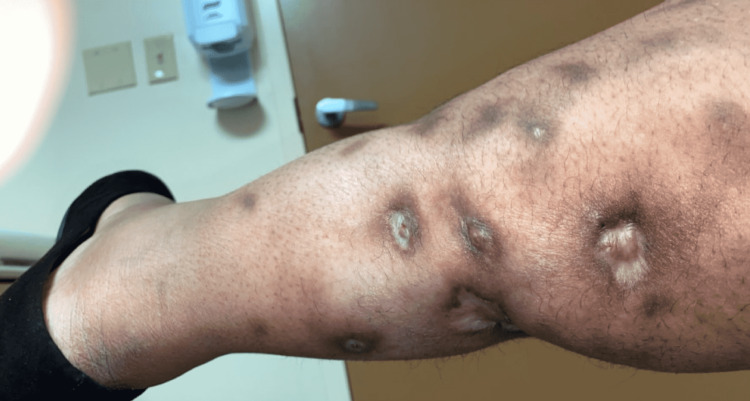
Posterior image of the right leg after initiating x2 doses of rituximab Lesions are starting to heal with noticeable scarring and have become less painful

Laboratory workup was again unremarkable for lupus anticoagulant, anti-cardiolipin antibodies, beta-2-glycoprotein antibody, protein C, and protein S activity. Steroids were discontinued, as we suspected it was worsening the lower extremity ulcers. The patient was given a trial of 25 grams of sodium thiosulfate (STS) intravenously, but she was intolerant to it due to severe gastrointestinal symptoms. Due to the presence of severe pain on palpation and numerous ulcerations, topical and intradermal methods of STS were deemed not feasible. Given the background of autoimmune connective tissue disorder and no evidence of renal failure, antiphospholipid syndrome, or use of warfarin, rituximab therapy was provided as a salvage effort. The patient received two doses of rituximab 1000 mg intravenously 14 days apart with gradual healing of the ulcers. She was maintained on two doses of rituximab 1 g every 14 days, followed by every six months for one year with excellent results (Figure 3).

## Discussion

Historically, calciphylaxis was viewed as a long-term complication of ESRD. Although rare, this complication can occur in patients with normal renal function, and the condition is termed NUC. The mortality rate is upward of 50%, mostly secondary to sepsis as the ulcerations serve as a nidus for infection and subsequent bacteremia [3]. Patients present similarly with painful skin lesions that are often indurated and hyperpigmented, typically involving the lower extremities.

Several disease states have been associated with NUC, including primary hyperparathyroidism (27.8%), cholangiocarcinoma, chronic myeloid leukemia (CML), melanoma and other malignancies (22.2%), alcoholic liver disease (16.7%), and connective tissue diseases (11.1%) [1]. Of note, Nigwekar et al. have reported that 61% of patients with NUC had concomitant steroid use, and protein C and S deficiencies were seen in 11% of patients. There are very few case reports describing the onset of NUC in patients with autoimmune disease who have been on chronic immunosuppression via corticosteroids [3]. In patients with autoimmune disease, poorly controlled disease progression has been theorized as a risk factor. Kusari et al. presented a case of a 60-year-old woman who still developed NUC despite the fact that her multiple autoimmune diseases including SLE were well-controlled [4]. Several risk factors and comorbidities associated with NUC have been summarized by Gomes et al. (Table 1).

**Table 1:** Nonuremic calciphylaxis - associated risk factors and comorbidities* *[5] PTH: parathyroid hormone

Risk factors and comorbidities [5]	
Disease states	Examples
Nonuremic chronic kidney disease	-
PTH abnormalities	Chronic hyper- and hypoparathyroidism
Vitamin D abnormalities	-
Osteoporosis and other conditions with bone mineral loss	-
Malignancy (solid and hematological)	Cholangiocarcinoma, endometrial adenocarcinoma, malignant melanoma, metastatic breast cancer, metastatic parathyroid carcinoma with primary hyperparathyroidism, multiple myeloma, chronic myelocytic leukemia
Autoimmune and granulomatous diseases	Systemic lupus erythematosus, rheumatoid arthritis, giant cell arteritis, sarcoidosis, Crohn’s disease
Other chronic inflammation states	Alcoholism, liver disease, aluminum toxicity
Prothrombotic conditions	Protein C and S deficiency, antithrombin III deficiency, cryofibrinogenemia, antiphospholipid antibody syndrome
Diabetes mellitus	-
Drugs	Corticosteroid use, warfarin, chemotherapy (cyclophosphamide, Adriamycin, fluorouracil)
Other	Infection, obesity, rapid weight loss, female gender

Our patient had multiple risk factors for NUC, such as female gender, obesity, superimposed infections, SLE, and chronic prednisone use. The diagnosis of NUC can be challenging since the differential for painful lower extremity nodules and ulcerations is extensive. Specifically in SLE patients, lupus panniculitis, otherwise known as lupus profundus, can present in a very similar fashion. NUC could be the end-stage progression of lupus profundus in an SLE patient. Skin biopsy remains the confirmatory testing of choice. Histological features remain similar with calcifications in the medial layer of arterioles and intimal fibrosis (Figure 2). The calcifications lead to endothelial dysfunction causing microthrombi to form. It is the thrombi that cause tissue ischemia leading to painful lesions and skin necrosis [1]. A prothrombotic state is currently considered as the main driver for calciphylaxis in nonuremic patients since the regulatory mechanisms regarding calcium, phosphate, and PTH remain intact [3]. It has been postulated that vascular calcification occurs from active cellular processes involved in biomineralization and the NF-kB pathway [5]. Thus, NF-kB inhibition could hold promising benefits in NUC but would require further investigation. Another theory holds that vascular smooth muscle cells transform into osteoblast-like cells that produce and deposit hydroxyapatite crystals within the vasculature, leading to calcium deposition, similar to ectopic bone formation in the vessel walls [6,7].

There are only a few case reports describing the onset of NUC in patients with autoimmune disease who have been on chronic immunosuppression via corticosteroids [3]. Our patient was on varying doses of prednisone for several years for SLE treatment. Wound care, pain control, and treating the underlying disease are the mainstay of therapy. Therapies aim to normalize mineral metabolism to reduce the serum concentration of calcium-phosphate byproducts and thereby prevent precipitation and calcification. Bisphosphonates, phosphate binders, cinacalcet, and STS have been used with variable success [3]. Still, much of the current treatments are extrapolated from the treatment of calciphylaxis patients. For example, the use of STS treatment in the treatment of NUC is still undergoing further investigation in phase 3 of the CALISTA Trial for the treatment of acute calciphylaxis patients with ESRD on hemodialysis [8].

In our case, the patient failed to respond to multiple therapies to control her SLE and was given salvage therapy with rituximab, a monoclonal antibody directed against the CD20 antigen on the surface of B-lymphocytes that activates complement-dependent B-cell cytotoxicity [3]. There is no data in the current literature about the specific use of CD20 inhibitors for the treatment of NUC. Rituximab works by causing B-cell depletion and was originally used for the treatment of non-Hodgkin's lymphoma. However, rituximab has also been shown to be effective in randomized controlled trials for rheumatoid arthritis, granulomatosis with polyangiitis, and other antineutrophil cytoplasmic antibody-associated vasculitides [9]. Rituximab is now increasingly being used for other autoimmune disorders as well. The efficacy of rituximab for autoimmune disorders is potentially attributed to a decrease in the rate of new plasma cell synthesis, as CD20+ B cells are required as an intermediary in this process [9]. Additionally, it is presumed that rituximab leads to a disruption in the role of B cells as antigen-presenting cells to T cells [9]. The loss of B cells from circulation is transient, usually lasting up to six months [9]. We started rituximab in our patient as salvage therapy and to discontinue high-dose prednisone, as we suspected that steroids were making her nonhealing ulcers worse.

## Conclusions

NUC is often a very debilitating and lethal condition with no known effective treatment. The disease is a diagnostic and therapeutic challenge with a high risk of septic complications. Due to the high rate of mortality associated with it, early recognition is imperative, and alternative therapeutic measures need to be considered in refractory cases. Wound care and treating the underlying condition remain paramount. This report highlights the successful use of B-cell depleting therapy with rituximab in a case of NUC refractory to conventional therapies in a patient with risk factors such as autoimmune disease, female gender, obesity, and chronic prednisone use. Further investigation is needed into the use of CD20 inhibitors for the treatment of NUC.
